# Eco-Friendly Nitrogen-Doped Graphene Preparation and Design for the Oxygen Reduction Reaction

**DOI:** 10.3390/molecules26133858

**Published:** 2021-06-24

**Authors:** Monica Dan, Adriana Vulcu, Sebastian A. Porav, Cristian Leostean, Gheorghe Borodi, Oana Cadar, Camelia Berghian-Grosan

**Affiliations:** 1 National Institute for Research and Development of Isotopic and Molecular Technologies, 67-103 Donat Street, 400293 Cluj-Napoca, Romania; monica.dan@itim-cj.ro (M.D.); sebastian.porav@itim-cj.ro (S.A.P.); cristian.leostean@itim-cj.ro (C.L.); gheorghe.borodi@itim-cj.ro (G.B.); 2 INCDO-INOE 2000, Research Institute for Analytical Instrumentation, 67 Donat Street, 400293 Cluj-Napoca, Romania; oana.cadar@icia.ro

**Keywords:** ball milling, functionalized graphene, oxygen reduction performance, machine learning, reverse engineering

## Abstract

Four N-doped graphene materials with a nitrogen content ranging from 8.34 to 13.1 wt.% are prepared by the ball milling method. This method represents an eco-friendly mechanochemical process that can be easily adapted for industrial-scale productivity and allows both the exfoliation of graphite and the synthesis of large quantities of functionalized graphene. These materials are characterized by transmission and scanning electron microscopy, thermogravimetry measurements, X-ray powder diffraction, X-ray photoelectron and Raman spectroscopy, and then, are tested towards the oxygen reduction reaction by cyclic voltammetry and rotating disk electrode methods. Their responses towards ORR are analysed in correlation with their properties and use for the best ORR catalyst identification. However, even though the mechanochemical procedure and the characterization techniques are clean and green methods (i.e., water is the only solvent used for these syntheses and investigations), they are time consuming and, generally, a low number of materials can be prepared, characterized and tested. In order to eliminate some of these limitations, the use of regression learner and reverse engineering methods are proposed for facilitating the optimization of the synthesis conditions and the materials’ design. Thus, the machine learning algorithms are applied to data containing the synthesis parameters, the results obtained from different characterization techniques and the materials response towards ORR to quickly provide predictions that allow the best synthesis conditions or the best electrocatalysts’ identification.

## 1. Introduction

Nitrogen-doped graphene (N-doped graphene, N-Gr), obtained by the substitution of carbon atoms from the graphene layers with nitrogen, possesses interesting structure and electronic, magnetic and optical properties [1,2,3]. Due to the atomic size similarity of the carbon and nitrogen atoms, N-doping of the graphene sheets can be easily produced by direct synthesis when chemical vapor deposition (CVD), segregation growth, solvothermal or arc-discharge approaches are considered, or by post-synthesis techniques such as thermal, plasma or hydrazine treatments [4]. Using these methods, several N-bonding configurations are attained in the N-doped graphene structure; the four common configurations are N graphitic (or quaternary N), N pyrrolic, N pyridinic and pyridinic N-oxide (pyridinic-N-O) [4]. Thus, by employing these techniques, the control of the doping process could be more or less addressed. In many cases, this control is still challenging and good results are even more important, especially when large-scale production and environmentally friendly methods are involved. The mechanical ball milling procedure is considered as a green and accessible approach for graphene preparation [5]. It has also been applied for N-doped graphene synthesis, being able to provide advanced electrocatalysts for fuel cell, supercapacitor or lithium-ion batteries [6,7,8,9]. In this context, a 11.4 at.% N-doped graphene has been prepared by ball milling of graphite with melamine [7].

In recent years, research aimed at the development of efficient and inexpensive oxygen reduction reaction (ORR) catalysts has involved the use of the N-doped graphene materials, coupled or not with some transitional metals, such as iron or cobalt [10,11,12,13]. Due to the electronic interaction between the lone pair electrons of nitrogen and the pi-electronic system of the graphitic structure, these materials exhibited electrocatalytic performance towards ORR that was comparable with the Pt/C catalyst [14]. Thus, N-doped graphene has received a lot of attention and has opened new insights in the fields of the energy application technologies [15].

The oxygen reduction reaction (ORR) is a kinetically slow process, requiring a transfer up to four electrons and four protons, and at least three intermediates are involved during the catalytic procedure [16]. The 4e^−^ reaction pathway is preferred to the 2e^−^ one yielding to water molecules, and it9 is considered a plausible pathway for ORR on doped graphene in both acid and alkaline media [17]. However, there are still debates related to the nature of the active sites for ORR contained in the nitrogen-doped carbon materials; some studies indicate the graphitic N, while others consider the pyridinic N or pyrrolic N as the active centers in the ORR process [18]. Moreover, different opinions are pointed out related to the significance of nitrogen percentages from N-doped materials onto their ORR catalytic performances; there are voices claiming improvements of catalytic activity with increases in the amount of nitrogen, while others consider the active center contents as being the key for catalytic performance [18].

In our research, we have performed a ball milling treatment in order to prepare nitrogen-doped graphene from graphite and melamine as nitrogen source. In addition to the advantage of obtaining large quantities of functionalized graphene-based materials, this protocol allows the development of materials by a green approach (a clean and safe chemistry), without the employment of polluting solvents. Four types of N-doped graphene with N wt.% between 8.34 and 13.1 have been obtained and their ORR performance has been evaluated using cyclic voltammetry and rotating disk electrode protocols. To determine the correlation of the ORR catalytic performance with the materials’ structure, we analysed the hybrid materials by different methods, including transmission and scanning electron microscopy (TEM, SEM), thermogravimetric measurements (TGA), elemental analysis, X-ray photoelectron spectroscopy (XPS), X-ray powder diffraction (XRD) and Raman spectroscopy. Moreover, to facilitate the materials’ design and to establish the best synthesis conditions, the machine learning algorithms have been trained on variables containing as input the data that combine both the experimental conditions and different characterization parameters, and as output the number of electrons transferred during the ORR process. The predicted results were then used to identify the optimization of material properties and the best synthesis conditions by applying the reverse engineering process.

## 2. Results and Discussion

### 2.1. Morphological, Thermal Stability and Chemical Characterization

#### 2.1.1. Morphological Analysis

**Figure 1:**
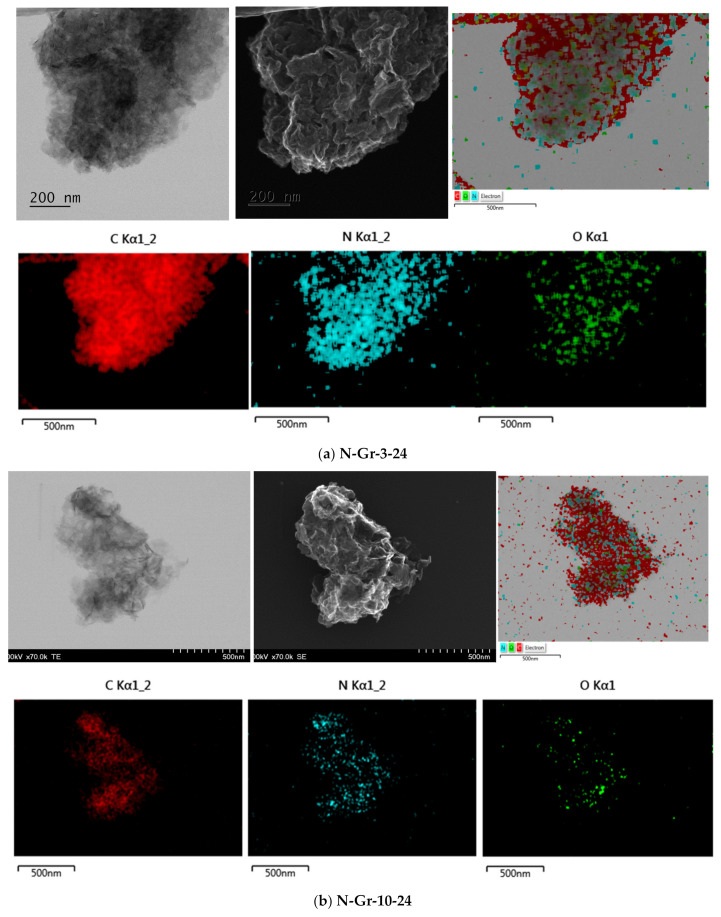

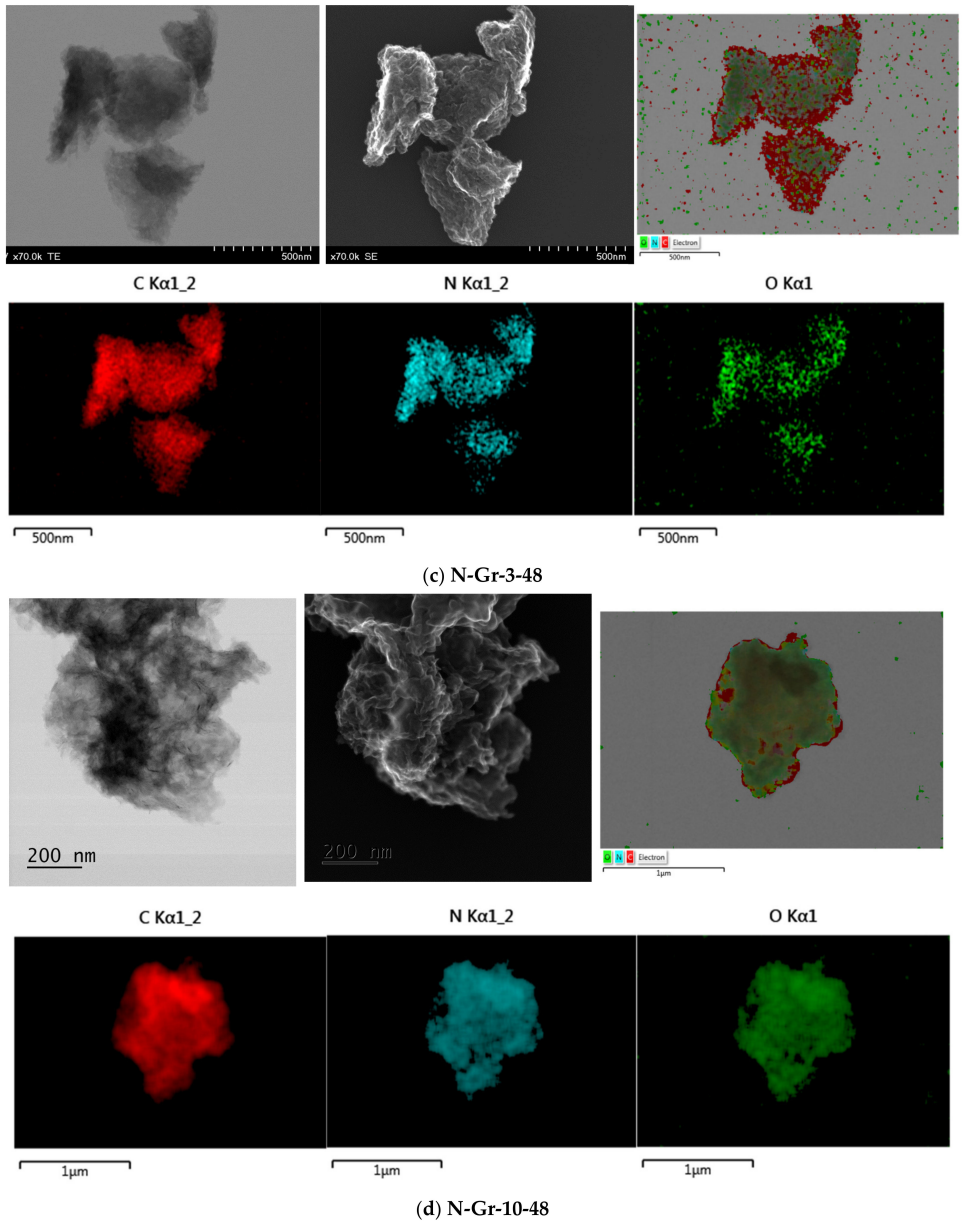
TEM, SEM and EDX elemental mapping for the (**a**) N-Gr-3-24; (**b**) N-Gr-10-24; (**c**) N-Gr-3-48; and (**d**) N-Gr-10-48.

Figure 1 shows the morphological characterization of the investigated materials. Thus, the TEM, SEM and compositional elemental mapping of the four N-doped graphene (N-Gr-3-24, N-Gr-10-24, N-Gr-3-48, N-Gr-10-48) are presented; the first number represents the amounts (g) of melamine used in the synthesis procedure, while the second one is the time (h) employed for grinding in the ball milling process (see Materials and Methods section).

These images indicate the nitrogen doping of the graphene and the presence of oxygen species on the graphene surface. Moreover, the graphene obtained by ball milling procedure appears to have a fluffy structure, very different from that synthetised by CVD or chemical methods [14,19,20] or the graphite structure (Figure 1 and Figure S1, Supplementary Materials). It is clear from these pictures that multilayer and crumpled graphene are synthetized by this method, suggesting the idea of multiple defective sites and edges on the graphene and explaining the high density of heteroatoms (nitrogen and oxygen) observed in the EDX elemental mapping of these hybrid nanomaterials (Figure 1).

#### 2.1.2. Thermal and Elemental Analyses

**Figure 2:**
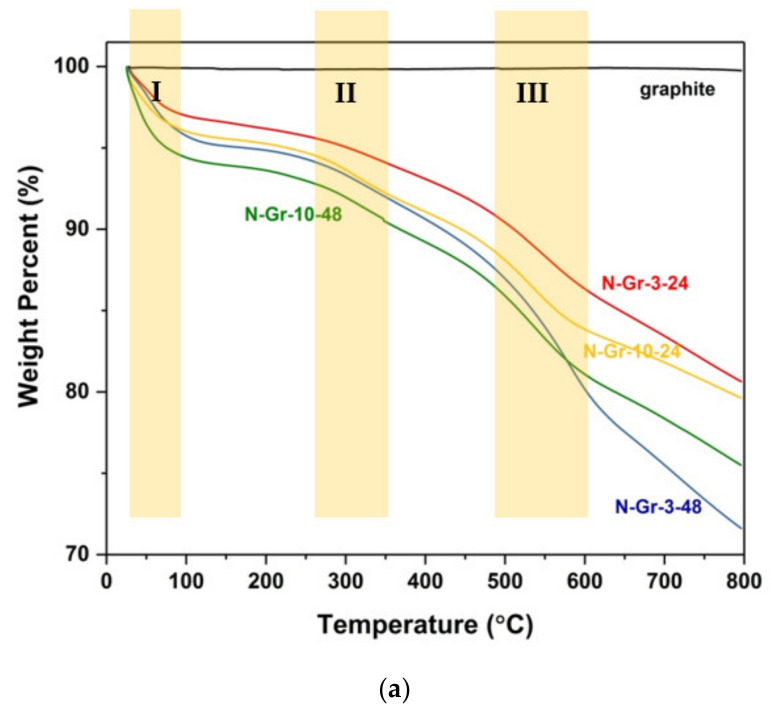

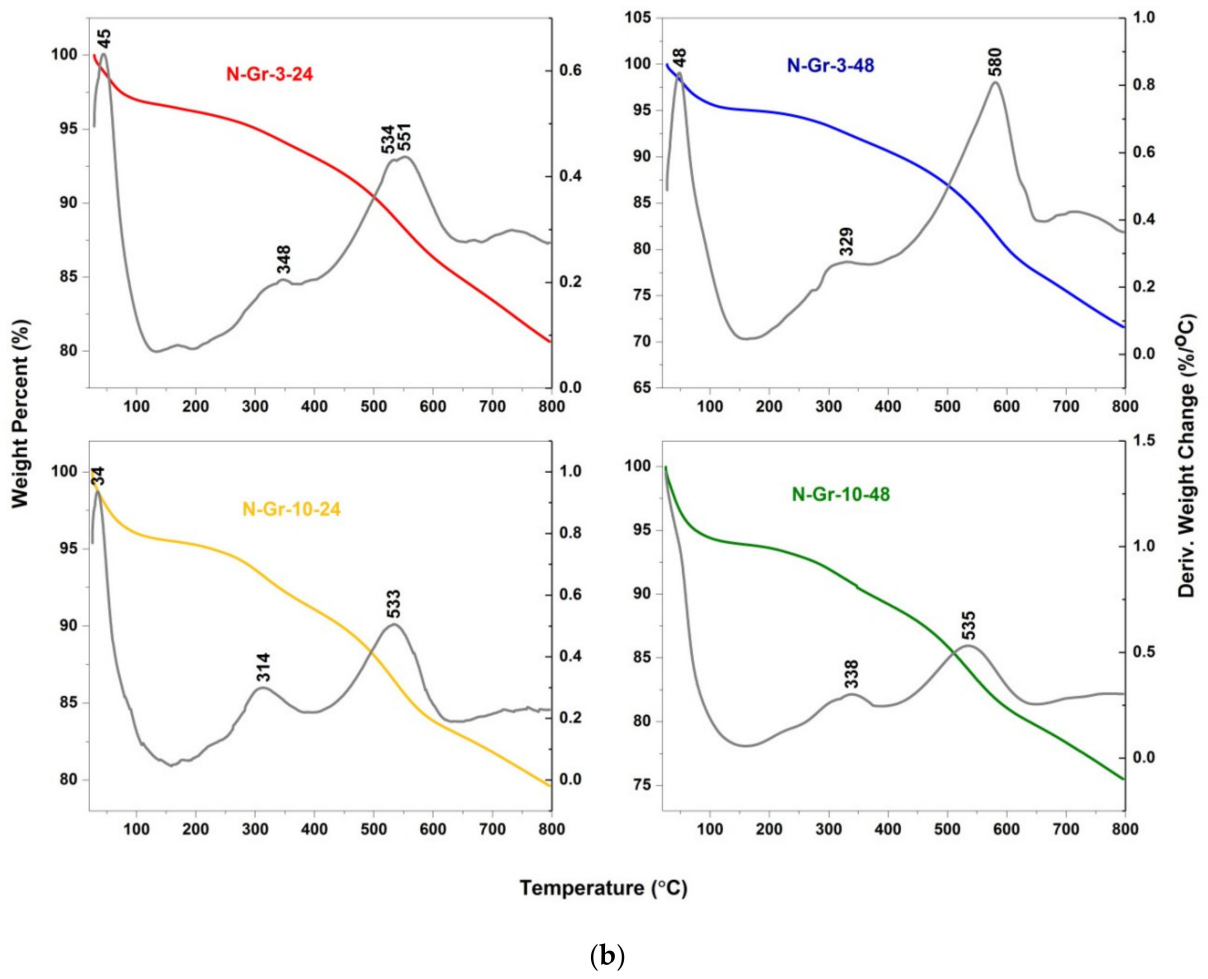
(**a**) Thermogravimetric (TG) and (**b**) derivative thermogravimetric (DTG) curves of the N-doped graphene materials carried out under the argon (Ar) atmosphere; the graphite was also added in the TG analysis to highlight the differences between it and the hybrid materials’ thermal stabilities.

A first idea about the functionalities found on the graphenes’ surface is given by the thermogravimetric analysis of nanocomposites under the argon atmosphere (Figure 2 and Table 1). The physical mixtures of the graphite–melamine 1:3 and 1:10 ratios have been also investigated by TGA analysis under the argon atmosphere (Figure S2); these results indicate a weight loss of 76.8% and 91.6%, respectively, corresponding to the melamine degradation (at about 326–329 °C).

For the hybrid materials, the TG and DTG curves, obtained under the argon atmosphere, show nearly no weight loss for graphite (as we already observed from the physical mixtures’ investigation, see Figure S2), while, for the N-doped materials, three separate regions can be observed (Figure 2). The first one corresponds to the adsorbed water content; it is situated until 100 °C and has a weight loss of about 3–5.7% (Table 1). The second weight-loss step, ranging in the temperature region of 125–360 °C, is most probably due to the intermolecular dehydration of the vicinal carboxylic or hydroxylic groups, yielding to most stable oxygen-compounds (lactones, anhydrides, ethers and carbonyls) and releasing the so-called “chemical water” [21]. A decarboxylation process of individual carboxylic groups, with the emission of CO_2_, can be also seen in this region [21]; the materials have a weight loss of about 2.5–4.4% in region II (Table 1). The third region, from 400 to 600 °C, can be associated with the decomposition of anhydrides and individual phenols and shows a weight loss of 7.3–13.9% (Table 1). Moreover, in addition to the previous steps, the functionalized material exhibits a continuous weight loss, reaching a total weight loss of about 19.4–28.4% (Table 1); this weight loss can be obtained from the decomposition of lactones or individual ethers [21].

The evolution of the nitrogen functionalities into the graphene structure must also be considered. Thus, based on the thermal stability of nitrogen groups [22], the decomposition of pyridinic N-O oxide in region II and the transformation of pyrrolic N into the pyridinic N in the temperature range of 600–800 °C [23] must be noted for this type of material. Finally, the residual carbon, calculated from the TGA measurements, shows a good correlation with the carbon elemental analysis (Table 1).

The authors assume that a small oxygen percentage from the four N-doped graphene could be from the graphite, but most of the oxygen content is from the water adsorbed on graphene during the material treatment (washing with hot water)—region I of the TGA—and from the O-functional groups that are formed during the grinding process, considering that the materials are obtained in air and not under argon or nitrogen atmosphere. According to the data from Table 1, N-Gr-3-48 is the material most exposed to the environmental conditions (oxygen and air humidity); it is obtained from a 1:3 ratio of graphite/melamine and after 48 h of grinding at 400 rpm.

#### 2.1.3. X-ray Photoelectron Spectroscopy (XPS) Investigation

**Figure 3:**
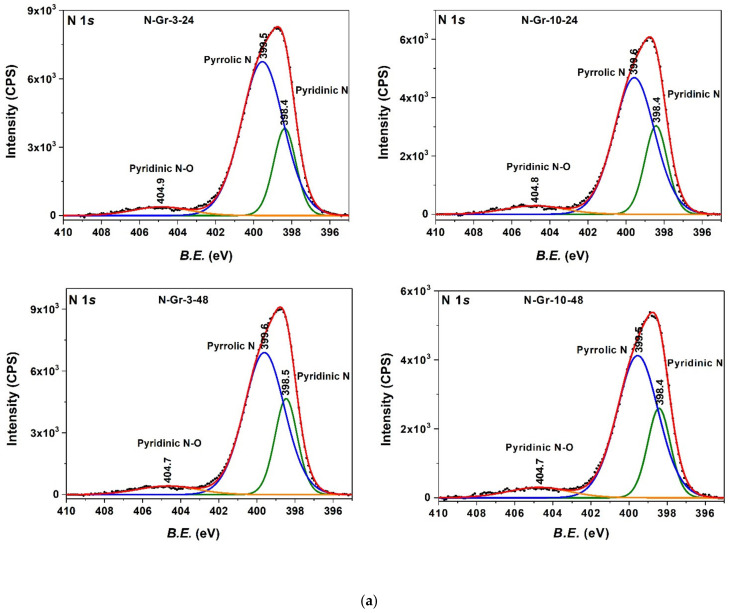

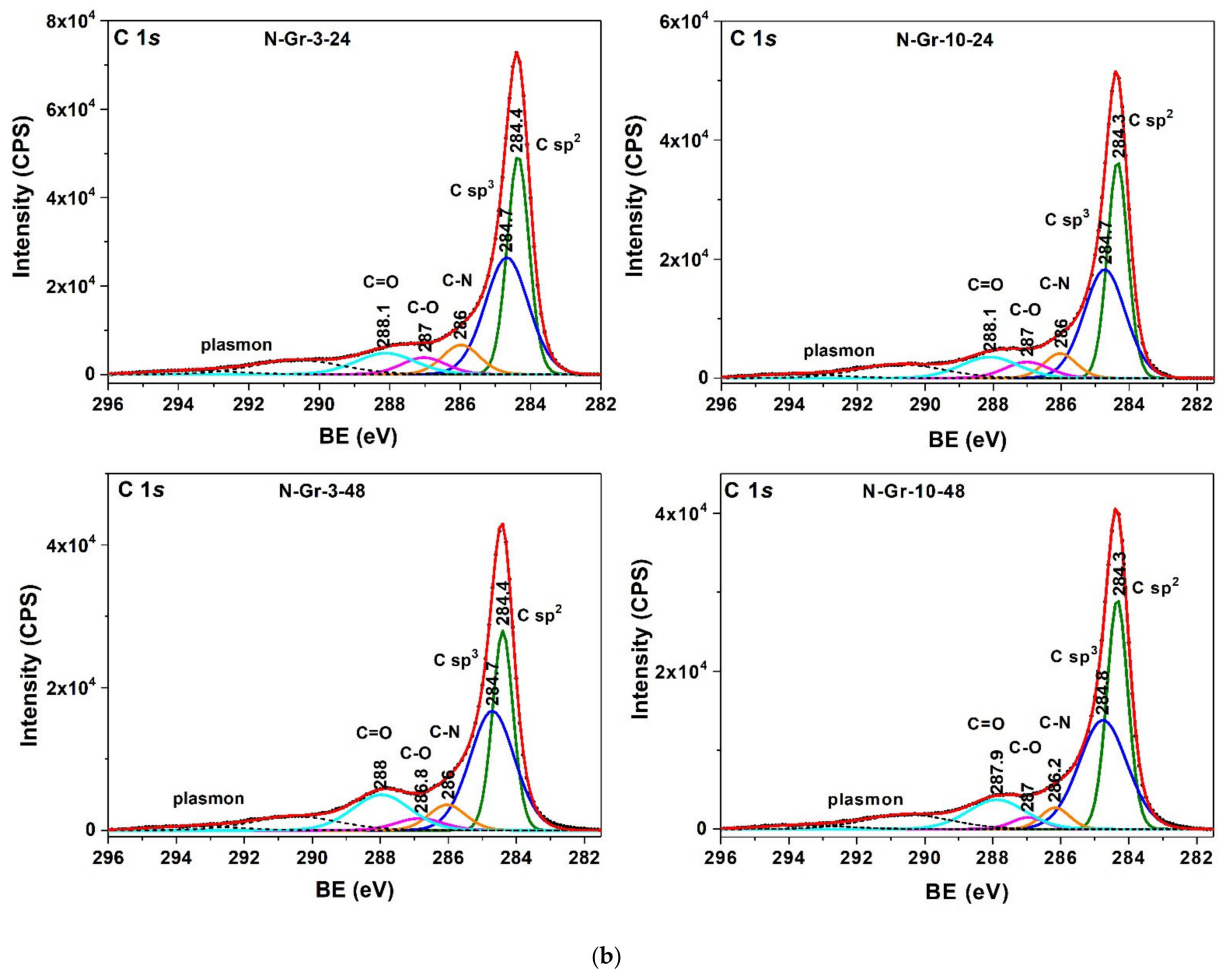
(**a**) N1s and (**b**) C1s XPS spectra analysis for the four types of N-doped graphene hybrid materials.

Further analysis of the N-doped graphene surfaces has been performed by X-ray photoelectron spectroscopy (XPS) and the results are presented in Figure 3 and Figure S3.

Three major nitrogen types—pyridinic-N 398.4 eV, pyrrolic-N 399.5 eV, and pyridine-N-oxide ~404.9 eV—have been identified from the XPS measurements [4,24]. The presence of pyridinic-N and pyrrolic-N, and the lack of graphitic-N, suggest the doping of the graphene layer at the edges or the structural vacancies. The comparison of the elemental composition (Table 1) and the XPS N-functional groups’ distribution (Figure S3) shows that no analogy can be established between the two analyses for the investigated materials. The inhomogeneity of the samples due to the N-doping at the edges and vacancies and the different natures of the two methods (total combustion of sample and surface analysis, respectively) are the main reasons for the observed variation. Regarding the distribution of the N-functional type, the pyrrolic-N fraction seems to have the highest value; the N-Gr-3-24 sample has the highest percentage of N-pyrrolic type, followed by the N-Gr-10-24 and N-Gr-10-48 ones (Figure S3).

The quality of the four materials has been investigated by considering the XPS percentages obtained for the C1s, in terms of the Csp^2^/Csp^3^ ratio and the content of oxygen functionalities from the graphene surface (Table S1). The results show a better quality for the carbon materials prepared by the 24 h ball milling procedure (the highest Csp^2^/Csp^3^ ratio and the lower quantity of oxygen functionalities on the graphene surfaces).

#### 2.1.4. X-ray Diffraction (XRD) Analysis

**Figure 4:**
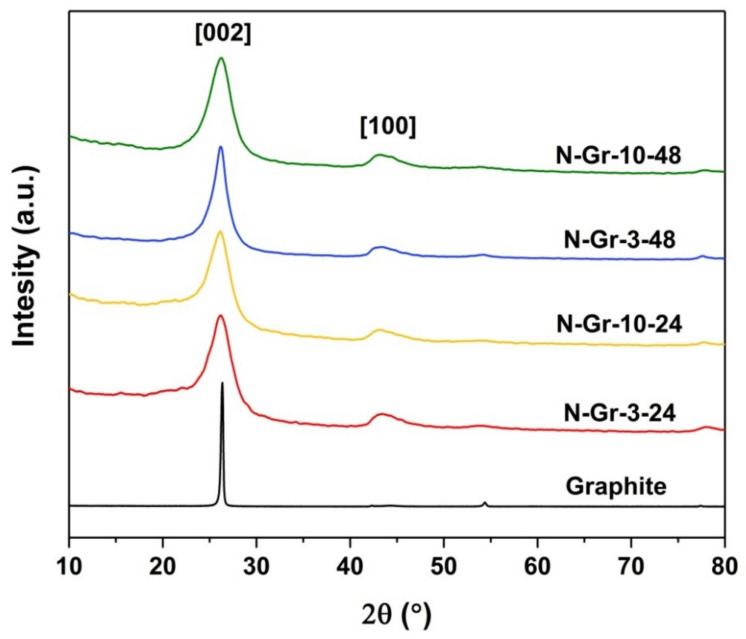
XRD spectra obtained for the graphite and the N-doped graphene materials.

The X-ray diffraction (XRD) patterns of the four N-doped graphene are presented in Figure 4 and Table S2. We observe a broadening of the peak at 2θ = 26°, corresponding to the [002] diffraction plane, for all the N-Gr-materials, indicating that the N doping of the graphite has been successfully induced by the ball milling process. According to Jeon et al. [25], the doping process occurs on the edges of the graphene. The N-doping process occurred on all the materials regardless of the milling time and the ratio between the components. The peak at about 2θ = 43° recorded for nitrogen-doped graphene corresponds to [100] reflection of the honeycomb structure; it is related to the sp^2^ hybridized carbons [26]. The main parameters calculated from the appropriate XRD results (Table S2) highlight the differences between the samples. The lowest 2θ and the highest FWHM parameters can be associated with the N-Gr-3-48 material; this sample possesses the lowest crystallite size and number of graphene sheets. Moreover, from all the N-doped graphene, the N-Gr-10-24 sample has the lowest FWHM, the highest crystallite size (except graphite), and the highest number of graphene layers.

According to these results, the number of graphene sheets depends on the quantity of melamine and the reaction time; when a large quantity of melamine is involved, a reaction time of 24 h is not enough to lead to low graphene layers (larger quantities of billing agent/reactant need more energy to accomplish the graphite exfoliation). When the reaction time is 48 h, the differences between the general parameters of the two composites are small (Table S2). The correlation of these results with those obtained by XPS investigations helps us to understand that a reaction time of 48 h provides, in fact, a deterioration of the graphene surface.

#### 2.1.5. Raman Spectroscopy Investigations

**Figure 5:**
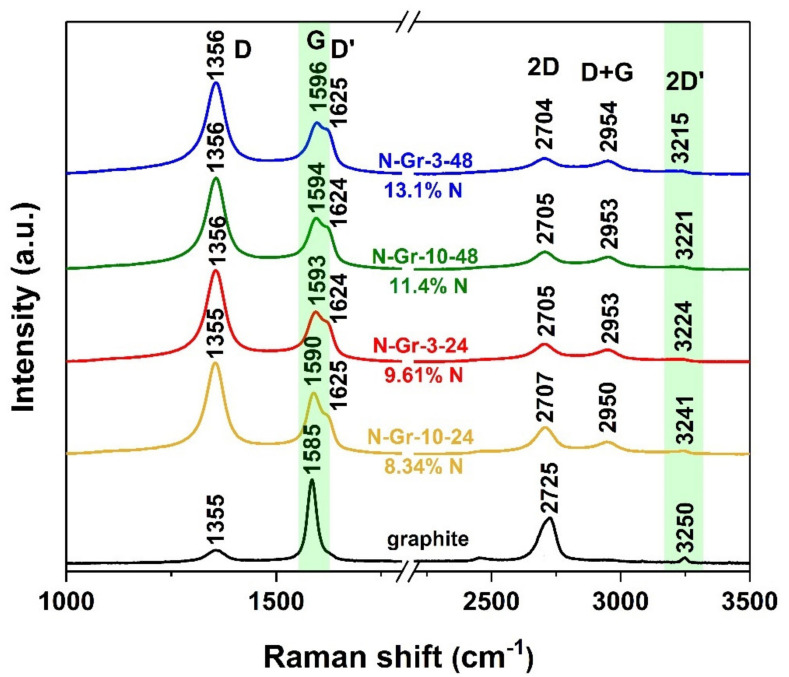
Raman spectra obtained for the N-doped graphene materials.

Figure 5 and Table S3 show the Raman spectra as well as different parameters obtained from these spectra; for an easy comparison, the N-doping concentrations of materials are also indicated. The band positions are obtained after the Lorentzian peaks’ deconvolution, while the N concentration values are taken from the elemental analysis.

The analysis of the Raman results of the graphite and the new prepared hybrid materials reveals a particular pattern under the 514.5 nm laser irradiation. The main features in the Raman spectrum of graphite are the D, G and 2D bands localized at 1355, 1585 and 2725 cm^−1^. Thus, the 1355 cm^−1^ peak, which is characteristic of polycrystalline graphite, can be attributed to a particle size effect and its intensity is inversely proportional to the crystallite size [27] (Figure 5 and Table S3). This band significantly increases in the N-doped graphene, indicating a reduction in their crystallite size, as is also revealed by XRD measurements (Table S2). The D peak is also activated by defects and its intensity increases in disordered graphene alongside with other two disorder peaks, D′ (about 1625 cm^−1^) and D + G (about 2950 cm^−1^) [28]. In Figure 5, the G and 2D′ bands’ regions are marked in green due to their shifting with the increase in N-doping percentage; thus, the G peaks shift to a higher frequency (from 1590 cm^−1^ to 1596 cm^−1^) as the N concentrations increase, and the 2D′ peaks show a down-shifting (from 3241 to 3215 cm^−1^) associated with the N content rising. The 2D peaks are located between 2707 and 2704 cm^−1^; however, the slight redshift of the 2D bands could not be directly associated with the increasing of the N amounts in the composite (Figure 5).

Looking to the data from Table S3, it seems that there is not a relationship between the I_D_/I_G_ ratio and the nitrogen content of the four hybrid materials. However, the ratio between I_D’_ and I_G_ (I_D′_/I_G_)—calculated for the four N-doped graphene materials—increases from 0.41 to 0.56, showing a better correlation with the N materials’ content than that of the I_D_/I_G_. Nevertheless, when attention is paid to the differences between all I_D’_/I_G_ values, we observe that these differences are not entirely connected with the concentrations’ differences (a 0.1 I_D′_/I_G_ variation for 1.27 N-concentrations’ difference for the first two materials, and a 0.03 I_D′_/I_G_ variation for 1.7 N-concentrations’ difference for the last two composites, see Table S3). A detailed evaluation of the XRD and Raman results highlights the reliance of the I_D’_/I_G_ ratio on the graphene crystallite size and the numbers of graphene layers, showing a good correlation of these data (Tables S2 and S3).

The intensity of the 2D peak is much higher for N-Gr-10-24, which has the lowest amount of nitrogen, leading to a larger value of the I_2D_/I_G_ ratio; the I_2D_/I_G_ values decrease with the increase in nitrogen contents in the materials and agree with the literature data [29].

The evaluation of the I_D_/I_D′_ ratio indicates values between 3.54 and 3.9, which means more defects in the boundaries. However, this variation cannot be linked only to the nitrogen amounts from the hybrid materials. The I_D_/I_D′_ values can be explained by considering all the defects from the materials, including the CO groups from the graphene surface (Tables S1 and S3).

Although the 785 nm laser excitation is not generally used for the graphene characterization, due to the presence of melamine in the two physical mixtures (graphite–melamine 1:3 and 1:10), their investigation requires that this wavelength be used. The obtained results are presented in Figure S4, highlighting the differences between the N-doped graphene and physical mixtures, and, at the same time, between the N-doped graphene and graphite.

### 2.2. ORR Performance Investigation

**Figure 6:**
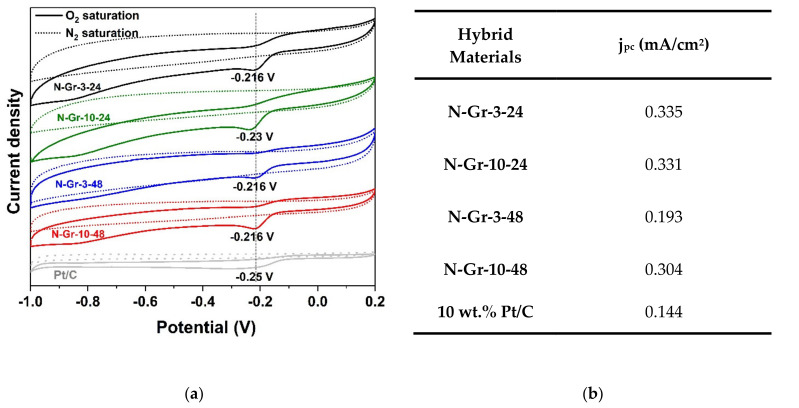
(**a**) Cyclic voltammograms (second cycle) of the hybrid materials/GC electrodes in N_2_- or O_2_-saturated 1M NaOH solutions at a scan rate of 50 mV/s; (**b**) ORR current densities.

**Figure 7:**
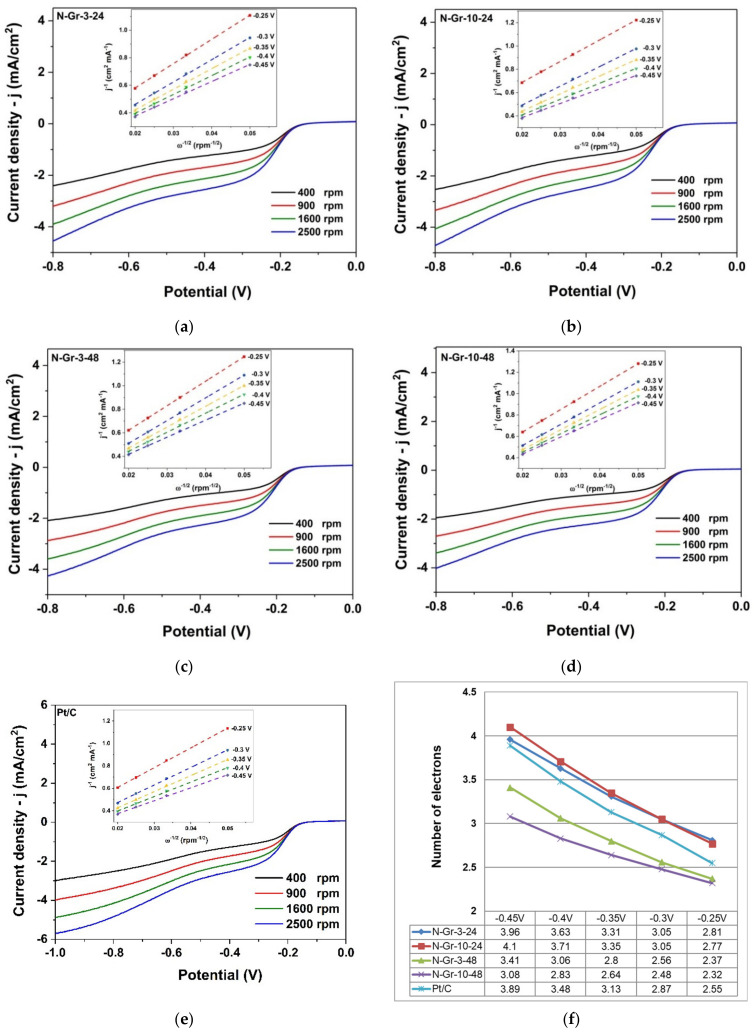
Current density-potential curves for ORR on N-doped graphene materials-coated glassy carbon electrodes: (**a**) N-Gr-3-24, (**b**) N-Gr-10-24, (**c**) N-Gr-3-48, (**d**) N-Gr-10-48, (**e**) 10 wt.% Pt/C; inset: the Koutecky–Levich plots for ORR. Spectra are recorded in O_2_-saturated 1M NaOH solution at various electrode rotating rates and a potential scan rate of 10 mV/s; (**f**) Number of electrons, at different potentials, transferred during the ORR process.

The electrocatalytic activity for ORR of the four N-doped graphene samples, prepared by the green procedure (ball milling of graphite and melamine), was tested in alkaline solutions of 1M NaOH. Figure 6 and Figure 7 contain the ORR results obtained for the investigated N-doped graphene materials.

Firstly, in order to explore the electrocatalytic properties of the N-doped graphene towards ORR, cyclic voltammetry (CV) measurements have been performed in N_2_- or O_2_-saturated 1M NaOH solutions at a scan rate of 50 mV/s using the N-doped graphene/glassy carbon (GC) modified electrodes; for comparison, the 10 wt.% Pt/C modified GC has been prepared and tested towards ORR in alkaline medium (Figure 6).

As can be seen in Figure 6a, the CV curves show an oxygen reduction peak at −0.216 V for N-Gr-3-24, N-Gr-3-48, N-Gr-10-48, and -0.230 V for N-Gr-10-24, demonstrating their potential as metal-free electrocatalysts for ORR in alkaline solution. The ORR current densities obtained from the second cycle are comparable for three of the four N-doped graphene materials investigated, indicating the following order of response: N-Gr-3-24 > N-Gr-10-24 > N-Gr-10-48 > N-Gr-3-48; all ORR current densities values are higher than the 10 wt.% Pt/C electrode response (Figure 6b). A higher response is obtained from the composite N-Gr-3-24, meaning that the better result is achieved when lower amounts of melamine and the lowest grinding time are employed in the synthesis procedure. *According to these results, neither the use of large amounts of melamine nor the increase in the reaction time is justified for ball milling preparation of efficient catalysts for oxygen reduction in alkaline media.*

A comparative study was also conducted using the rotating disc electrode (RDE) at different rotating rates from 400 to 2500 rpm on the appropriate N-doped graphene/glassy carbon (GC) (Figure 7). These experiments were performed to determine the number of electrons transferred in the electrocatalytic process. The insets from Figure 7a–d represent the Koutecky–Levich plots (j^−1^ vs. ω^−1/2^) at different potentials and show clear linearity. From the Koutecky–Levich (K–L) equation, presented below, the number of electrons (n) transferred in the ORR process is evaluated and presented in Figure 7f.

$$\begin{array}{c} \frac{1}{j}=\frac{1}{j_{l}}+\frac{1}{j_{k}}=\frac{1}{B\omega^{1/2}}+\frac{1}{j_{k}}\\ B=0.2nFC_{O_{2}}D_{O_{2}}^{2/3}\nu^{-1/6} \end{array}$$

*j*—measured current density; *j_l_*—diffusion-limiting current density; *j_k_*—kinetic-limiting current; *ω*—electrode rotation rate (rpm); *F*—Faradaic constant (96,485 C/mol); *C_O2_*—O_2_ concentration in 1M NaOH solution (0.843 × 10^−6^ mol/cm^3^); *D_O2_*—O_2_ diffusion in 1M NaOH solution (1.43 × 10^−5^ cm^2^/s); *n*—number of electrons transferred in the ORR process; *ν*—kinematic viscosity of the electrolyte (1.13 × 10^−2^ cm^2^/s) [30,31,32].

Based on the K–L equation, there is a variation of the numbers of electrons transferred with the potentials, indicating a complex mechanism. The best results are observed for N-Gr-3-24 and N-Gr-10-24, of about 3.96 and 4.1 at −0.45 V, which are close to the theoretical value of Pt/C (4 electrons). These hybrid materials appear to be more efficient for ORR compared to N-Gr-3-48 and N-Gr-10-48 (numbers of electrons transferred: 3.41 and 3.08 at −0.45 V).

It is clear that the changes occurring in the materials during the additional 24 h of the synthesis process are determinant for their ORR behaviours (Figure 6 and Figure 7). For the ORR process in alkaline media, two mechanism types can be considered involving four (4 or 2 + 2) electron or two (2) electron pathways [17]. If, for the materials prepared within 24 h, the four-electron pathway is foreseen, in the case of the last two materials (48 h synthesis time), there is an alteration in their response for ORR; most probably, for these composites, the two-electron pathway becomes more significant in the ORR process (Figure 7f). Moreover, the kinetic current densities, calculated from the Koutecky–Levich plots, were found to be 6.98 mA/cm^2^ for 10 wt.% Pt/C and 7.84 mA/cm^2^ for N-Gr-3-24, suggesting a better response towards ORR of the N-doped graphene material.

The long-term stability of the four nitrogen doped materials and Pt/C was investigated using cycling voltammetry (Figure S5). The catalytic activity retained after 400 cycles was found to be 73.4% for N-Gr-3-24, 70.7% for N-Gr-10-24, 64% for N-Gr-3-48, 36.6% for N-Gr-10-48 and 52.7% for Pt/C.

The catalysts’ tolerance towards CO poisoning was also investigated using CO stripping voltammetry. CVs were recorded in N_2_ and CO saturated 1M NaOH electrolyte, respectively (Figure S6). In the case of nitrogen doped graphene, no significant modification of the CVs’ profile was observed, while, for the Pt/C, two oxidation peaks appeared at −0.4 and −0.31 V vs. Ag/AgCl, showing a poisoning effect.

A comparison of the present work with some previous literature results, obtained by testing various nitrogen-doped graphene towards ORR in alkaline conditions, indicates a similar electrocatalytic behavior with a large electron transfer number (almost 4) and a high current density (4.7 mA/cm^2^) (Table 2).

To summarize, the hybrid materials’ response for the ORR seems to be strongly influenced by the quality of graphene structure (lower quality graphene materials show smaller peak current densities and the lowest number of electrons transferred during the ORR process, promoting, most probably, the hydrogen peroxide formation).

### 2.3. Machine Learning and Reverse Engineering for Materials Properties Optimization

Looking to the electrochemical response towards ORR of the investigated materials and analysing these results by comparison with the results obtained from different characterization techniques, some questions need to be addressed: have we tested the best materials for ORR or is it possible to find an N-doped material, prepared by ball milling procedure, with improved electrochemical properties for our study?

**Figure 8:**
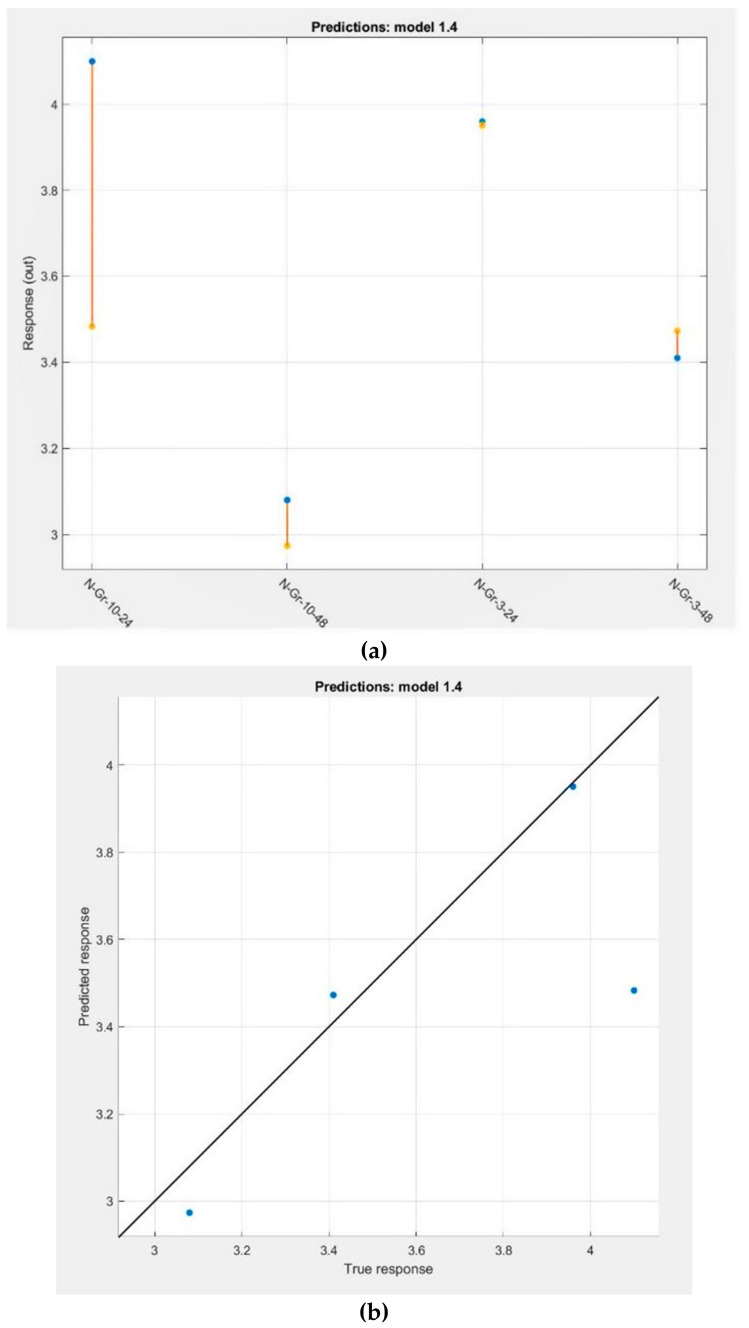
(**a**) True and predicted responses regarding the number of electrons transferred during the ORR process and the appropriate errors for the four investigated materials; (**b**) the predicted vs. actual plot for the Stepwise Linear Regression model.

K. Min et al. [38] have already proved the efficiency of machine learning (ML) techniques to assist the optimization of electrochemical properties of the cathode materials. Thus, by applying the ML algorithms and reverse engineering, ideal synthesis parameters are successfully proposed for the Ni-rich cathode materials optimization process. In these conditions, we decided to test the ML algorithms implemented in Matlab R2017b to construct a prediction model for the number of electrons transferred during the ORR process (output variable) by considering the experimental and measured parameters (input variables) presented in Table S4. The regression learner app was employed to obtain the predicted model and all 19 types of ML regression algorithm were trained for the prediction. After the training, the best model was identified and exported in the workspace. Among the regression models, the best RMSE (0.31449) and R^2^ (0.67) values were found for the Stepwise Linear Regression model. Figure 8 contains the Stepwise Linear Regression model results when the 4 fold cross-validation was used for training. The true and predicted responses regarding the number of electrons transferred during the ORR process and the appropriate errors for the four investigated materials are presented in Figure 8a, while the predicted response of the model is plotted against the perfect prediction in Figure 8b. From the figure, it is evident that the best predicted number of electrons transferred in the ORR process is obtained for the N-Gr-3-24 material (almost the same number, Figure 8a; point lies on the diagonal line which represents the perfect prediction line, Figure 8b) and the worst prediction is found for the N-Gr-10-24 composite (predicted number of electrons is much lower than the experimental value and the prediction error line is very large, Figure 8a; the experimental point corresponding to the 4.1 electrons is scattered at a significant distance from the prediction line, Figure 8b). These results explain the R^2^ value obtained for the model.

The predicted values of the numbers of electrons have been further employed in the reverse engineering process to establish the best synthesis conditions or the best material characteristics that should be obtained if we wish to improve the materials’ performances towards ORR. Thus, the inverse prediction function (***invpred***) has been applied to the predicted values, and to the most significant variables, when the number of electrons transferred during ORR is four.

Firstly, we applied the reverse engineering process for the synthesis parameters; our data make it possible to obtain perspective for the reaction time, but not for the melamine amounts. We continued with the most significant variables, the material characteristics that could provide information regarding the materials’ properties: the nitrogen doping percentage, the pyridinic and pyrrolic N content, the quality of graphene (Csp^2^/Csp^3^ ratio, C-O or C=O amounts from the graphene surface), the FWHM of the XRD peak, and the I_D′_/I_G_, I_2D_/I_G_ and G peak positions from Raman measurements. The results are presented in Table S4—prediction column; it seems that the synthesis reaction time needs to be extended to 25 h to provide the optimal number of electrons (four) transferred during the ORR process. The predicted parameters could be used as indications to guide the investigation of the structure–ORR response relationship, and could be involved in understanding how the materials can be modified for a better response.

## 3. Materials and Methods

### 3.1. Synthesis Section

Nitrogen doped graphene was prepared by grinding the graphite carbon rod sieved into a powder (99.9% Merck, Darmstadt, Germany) and 2,4,6-triamino-1,3,5-triazine (Merck, Hohenbrunn, Germany), further denoted as melamine, in a planetary ball milling machine (PM 400, Retsch Inc., Düsseldorf, Germany).

During the grinding process, a high amount of energy was released and activated the reactants, thus making the doping process possible. Therefore, the nitrogen atoms from the melamine could be introduced in the graphite network, yielding to the nitrogen functionalized material. Thus, the synthesis was conducted at a temperature much lower than the temperature commonly used for chemical synthesis.

Our process was performed at room temperature. The weighted graphite and melamine were transferred to a stainless-steel grinding jar with grinding balls (10 mm diameter, 30 balls) made of stainless steel. Two ratios between graphite and melamine were chosen: 1:3 and 1:10; in addition, two reaction times of 24 and 48 h were investigated.

Briefly, 1 g of graphite and the appropriate amounts of melamine were loaded in the milling bowl, sealed by fixing in the planetary ball-milling machine and grounded at 400 rpm for 24 h or 48 h, respectively. The obtained materials are denoted as N-Gr-3-24, N-Gr-3-48, N-Gr-10-24, N-Gr-10-48, in agreement with the ratios and reaction times employed in the synthesis procedure. Afterward, the materials were washed thoroughly with hot water to remove the un-reacted melamine.

### 3.2. Characterization

Transmission and scanning electron microscopy (TEM and SEM) were realized on a Hitachi HD 2700 (Hitachi, Tokyo, Japan) equipped with energy dispersive X-ray analysis (EDX).

Thermogravimetry measurements were carried out in argon using TA Instruments SDT Q 600 equipment (TA Instruments, New Castle, DE, USA) in the temperature range from 25 to 800 °C, with a heating rate of 10 °C min^−1^.

A Flash 2000 CHNS/O analyzer (Thermo Fisher Scientific, Waltham, MA, USA) analyzer was used to perform elemental analysis of the N doped material.

For X-ray Photoelectron Spectroscopy (XPS), a SPECS XPS (SPECS, Berlin, Germany) equipped with an Al/Mg dual-anode X-ray source, PHOIBOS hemispherical energy analyzer, 150 2D CCD, and a multichanneltron detector, were employed. The spectra were recorded for nitrogen, carbon, and oxygen elements by performing multiple scans with high resolution. Data analysis and curve-fitting were performed by employing a Gaussian–Lorentzian product function and a nonlinear Shirley background subtraction. The high-resolution spectra were deconvoluted into the components corresponding to different types of bonds.

The X-ray powder diffraction (XRD) analyses were recorded by using a Bruker D8 advanced diffractometer (Bruker, Karlsruhe, Germany) with Cu Kα1 radiation, operating voltage 40 kV, and current 40 mA. The diffraction patterns were measured in the range 15° < 2*θ*< 85° with a step size of 0.01°/s.

The Raman spectra were obtained using a JASCO NRS-3300 Raman Spectrometer (Jasco, Tokyo, Japan) with a CCD detector and excitation at 514.5 nm from an argon-ion laser. An Olympus objective UMPLFL 100X, 600 L/mm grating, 0.1 × 6mm slit, and the wavenumber centered at 2300 cm^−1^ were employed to record the spectra in the range of 73–4200 cm^−1^. Each spectrum was collected with an exposure of 100 s and a minimum of three scans; the data were analyzed using OriginPro 2017 software.

Electrochemical investigations, cyclic voltammetry (CV), rotating disk electrode voltammetry (RDE) and CO stripping voltammetry were performed on Autolab Potentiostat/Galvanostat 302N (Metrohm Autolab, Utrecht, The Netherlands), controlled by the Nova 1.11 software. The three-electrode system employed a graphene-based electrode (area 0.07 cm^2^) as the working electrode, Ag/AgCl (KCl, 3M), and platinum plate electrodes as the reference and auxiliary electrodes; all the measurements were realized in N_2_- or O_2_-saturated 1M NaOH solutions. For the RDE measurements, the spectra were recorded in O_2_-saturated 1M NaOH solution at various electrode rotating rates (400 to 2500 rpm) and a potential scan rate of 10 mV/s. The procedure for CO stripping experiments employed the following steps: (i) N_2_ was bubbled on the electrolyte (1 M NaOH) at a constant potential of −0.9 V vs. Ag/AgCl for 30 min, (ii) the gas was switched to CO and purged at the same potential for 30 min, (iii) the gas was switched back to N_2_ for 30 min, keeping the potential at −0.9 V vs. Ag/AgCl to remove the excess of CO gas from the electrolyte. The CO stripping voltammograms were recorded by cycling the potential between −1.0 and 0.2 V vs. Ag/AgCl with 20 mV/s scan rate.

### 3.3. Modified Electrode Preparation

The glassy carbon electrode (GC) having a diameter of 3 mm was used for modified electrode preparation. Before any treatment, the glassy carbon electrode was mechanically polished with chromium (III) oxide powder. An electrochemical treatment was also applied in 0.5 M H_2_SO_4_ by cycling the potential between −1.0 and +1.0 V vs. Ag/AgCl with a 50 mV/s scan rate. The modified electrodes were obtained as follows: 0.5 mg of the appropriate materials (N-doped graphene, 10 wt.% Pt/C) were suspended in 1 mL dimethylformamide and sonicated for 3 min using a finger sonicator (Sonics Vibra Cell, Newtown, CT, USA, 500 W, 20 kHz, ampl. 30%). Afterwards, 20 µl from these suspensions were drop-casted on the clean GC electrode and dried at room temperature.

### 3.4. Machine Learning and Reverse Engineering Investigations

Machine learning studies were performed using the Regression learner app implemented in MATLAB R2017b (MathWorks, Natick, MA, USA). The best predicted model was exported in the Matlab workspace and the predicted results were then used in the reverse engineering process; thus, the inverse prediction function (***invpred***) was applied to the predicted results, and the most significant variables, when the number of electrons transferred during ORR was 4.

## 4. Conclusions

We have prepared, by a green and easily scalable method, four types of N-doped graphene materials with a nitrogen content ranging from 8.34 to 13.1 wt.%. These materials have been tested towards ORR by cyclic voltammetry and rotating disk electrode protocols and their electrochemical responses have been compared with the results obtained from the surfaces and chemical investigation techniques.

Thus, morphological, thermal, elemental analyses and XPS investigations have been used to obtain data about the materials’ properties. Moreover, as a rapid screening for the performance of N-doped graphene in the ORR process, we showed that the correlation between the XRD and Raman spectroscopy methods can be effective. These methods give valuable information about the graphene crystallite size and number of graphene sheets, while some parameters attained from Raman data can be correlated with the nitrogen contents and crystallite size/number of graphene layers. Thus, the G peaks shift to higher frequencies when the N concentrations increase in the materials, the I_D′_/I_G_ and I_2D_/I_G_ ratios exhibit a good correlation with the nitrogen content and the crystallite size/number of graphene sheets, while the I_D_/I_D′_ values are similar with the current density responses obtained for the investigated materials.

By combining the synthesis parameters, the data obtained from different characterization techniques and the materials response towards ORR, we tested the possibility of using the machine learning algorithms for the optimization of synthesis conditions and prediction of the best structure parameters. The regression learner and reverse engineering methods have been employed to obtain these predictions. Our results highlight the advantage of using machine learning for the optimization of synthesis conditions and the design of materials.

## Figures and Tables

**Table 1:** Weight loss percentage (%) from TGA in Ar and elemental analysis of N-doped graphene.

N-Doped Graphene	TGA/Weight Loss %					Elemental Analysis/Weight %			
	Regions				Residual C	N	O	C	H
	I	II	III	Total till 800 °C					
N-Gr-3-24	3	3.2	7.3	19.4	80.6	9.61	9.31	80	1.08
N-Gr-10-24	4	3.6	8.7	19.8	80.2	8.34	9.43	81.3	0.93
N-Gr-3-48	4.6	2.5	13.9	28.4	71.6	13.1	12.98	72.5	1.42
N-Gr-10-48	5.7	4.4	8.9	24.5	75.5	11.4	7.34	80.2	1.06

**Table 2:** Electrochemical performance of different N-doped graphene towards ORR in alkaline media.

Samples	Catalyst Loading (mg/cm^2^)	Onset Potential	Current at 1600 rpm/ Potential (mA/cm^2^)	Electron Transfer Number
N-Gr-3-24 (this work)	0.14	−0.16 V vs. Ag/AgCl	4.7/−1 V vs. Ag/AgCl	3.96
N-aGS-800 [33]	0.18	0.03 V vs. Ag/AgCl	5.2/−1 V vs. Ag/AgCl	3.99
N-doped graphene (NG/Fe_5.0_) [11]	0.05	−0.04 V vs. Ag/AgCl	3.8/−1 V vs. Ag/AgCl	3.91
N-doped graphene [34]	0.152	0.82 V vs. RHE	4.4/0 V vs. RHE	3.9
N-doped graphene [35]	0.107	0.98 V vs. RHE	3.9 at 1500 rpm/ −0.2 V vs. RHE	3.9
N-doped reduced graphene oxide (n-RGO-850 °C)[36]	1.02	−0.15 V vs. Ag/AgCl	5.6/−1 V vs. Ag/AgCl	3.35
3D-NB-doped graphene [37]	0.082	−0.06 V vs. Ag/agCl	6/−0.9 V vs. Ag/AgCl	3.8

## Data Availability

The data presented in this study are available on request from the corresponding authors.

## Supplementary Materials

Figure S1: (a) TEM and (b) SEM images for graphite, Figure S2: TGA data of (a) 1:3 and (b) 1:10 graphite–melamine mixtures obtained under the argon atmosphere, Figure S3: (a) elemental (wt.%) and (b) N-functional groups’ distribution in the four types of N-doped graphene hybrid materials, Table S1: carbon types distribution (at%) obtained from XPS, Table S2: XD parameters obtained for the graphite and N-doped graphene materials, Figure S4: Raman spectra (excitation 785 nm) of the graphite, melamine, 1:3 and 1:10 graphite–melamine mixtures and N-doped graphene materials obtained by ball milling procedure, Table S3: Raman parameters obtained for the N-doped graphene materials, Figure S5: evaluation of the catalytic activity retention (%) over 400 cycles of the (a–d) N-doped graphene/GC electrodes, respectively (e) Pt/C/GC electrode in O2-saturated 1M NaOH solutions at a scan rate of 50 mV/s, Figure S6: CO stripping voltammograms of (a–d) N-doped graphene/GC electrodes, respectively (e) Pt/C/GC electrode in 1M NaOH, Table S4: Input and output variables used for ML investigations and the obtained prediction for a number of 4 electrons transferred during the ORR process.

## Sample Availability

Samples of the compounds are available from the corresponding authors.
