# Supplementary material for: Eco-Friendly Nitrogen-Doped Graphene Preparation and Design for the Oxygen Reduction Reaction
Source: Molecules. 2021 Jun 24;26(13):3858. doi: 10.3390/molecules26133858 (PMC8270343; doi:10.3390/molecules26133858)
Supplement: Supplementary file 1 [file molecules-26-03858-s001.zip › molecules-1271254-supplementary.pdf]

molecules

Supplementary Materials

Eco-Friendly Nitrogen-Doped Graphene Preparation and Design for the Oxygen Reduction Reaction

Monica Dan [1], Adriana Vulcu [1,*], Sebastian A. Porav [1], Cristian Leostean [1], Gheorghe Borodi [1], Oana Cadar [2] and Camelia Berghian-Grosan [1,*]

[1] National Institute for Research and Development of Isotopic and Molecular Technologies, 67-103 Donat Street, 400293 Cluj-Napoca, Romania; monica.dan@itim-cj.ro (M.D.); sebastian.porav@itim-cj.ro (S.A.P.); cristian.leostean@itim-cj.ro (C.L.); gheorghe.borodi@itim-cj.ro (G.B.)

[2] INCDO-INOE 2000, Research Institute for Analytical Instrumentation, 67 Donat Street, 400293 Cluj-Napoca, Romania; oana.cadar@icia.ro

* Correspondence: adriana.vulcu@itim-cj.ro (A.V.); camelia.grosan@itim-cj.ro (C.B.-G.)

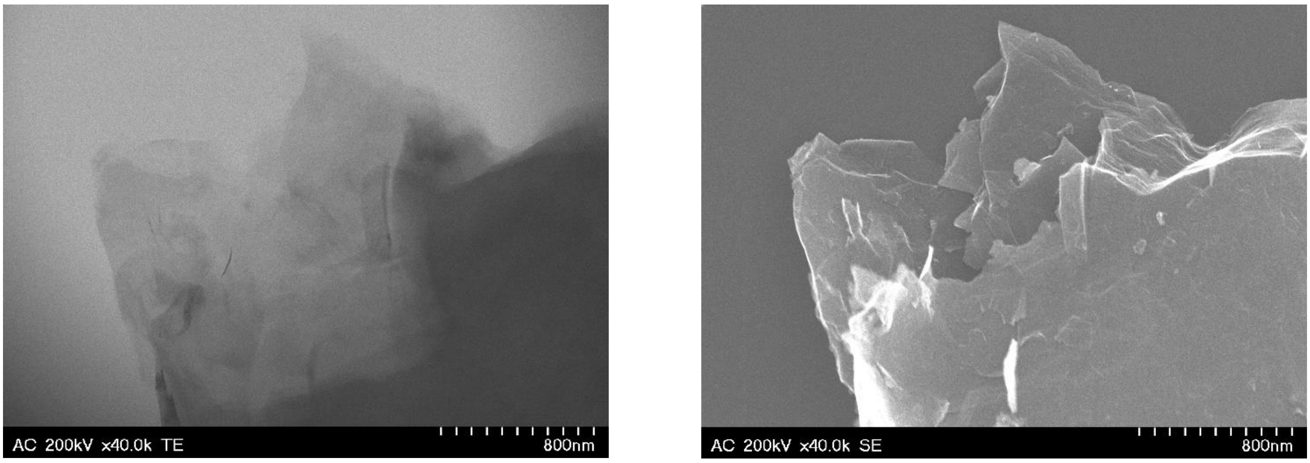

(a) **(b)**

Figure S1. (a) TEM and **(b)** SEM images for graphite.

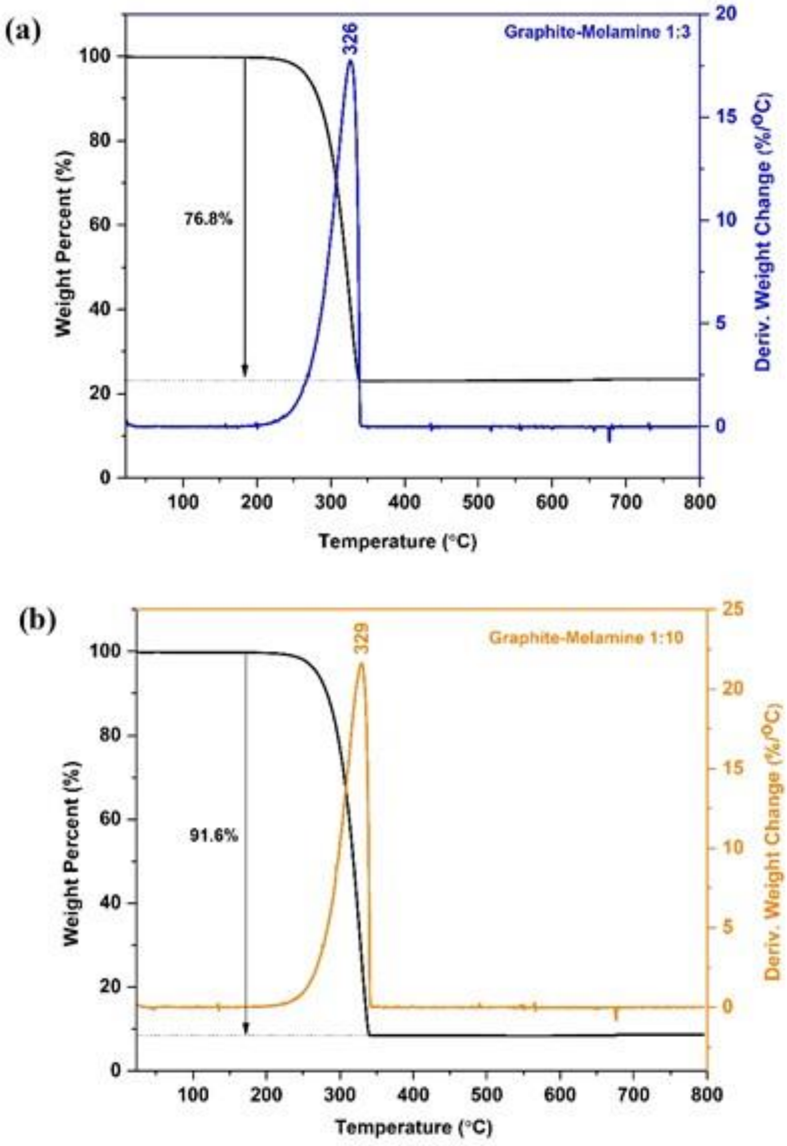

Figure S2. TGA data of **(a)** 1:3 and **(b)** 1:10 graphite–melamine mixtures obtained under the argon atmosphere.

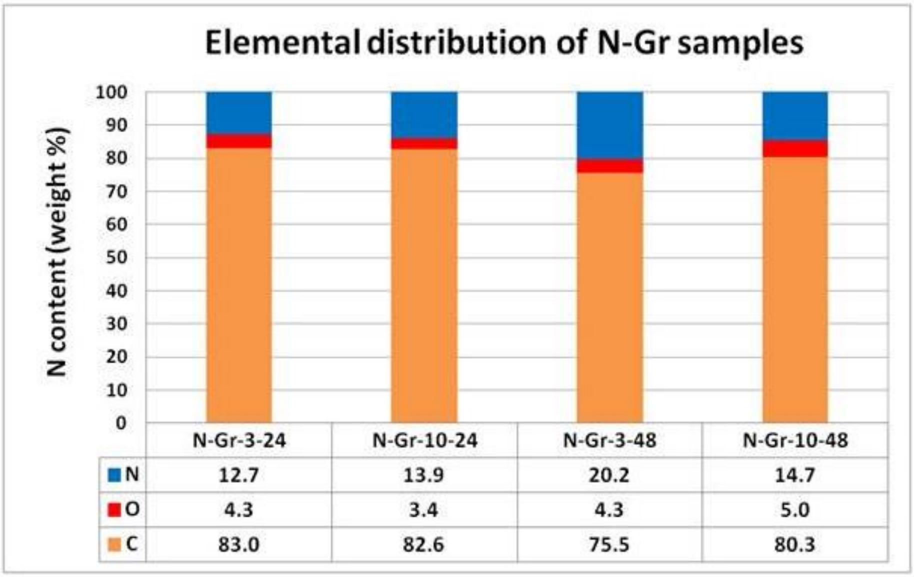

(a)

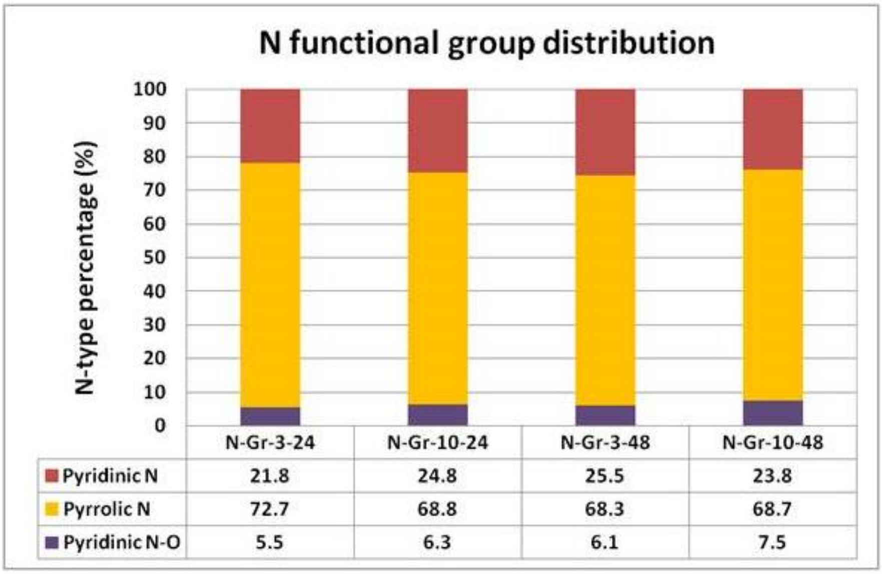

(b)

Figure S3. (a) Elemental (wt.%) and **(b)** N-functional groups' distribution in the four types of N-doped graphene hybrid materials.

Table S1. Carbon types distribution (at%) obtained from XPS.

N-doped Graphene	Csp^2	Csp^3	C-N	C-O	C=O	Csp^2/Csp^3
**N-Gr-3-24**	36.8	39.2	8.5	5.8	9.8	0.94
**N-Gr-10-24**	37.7	38.8	6.8	6.2	10.5	0.97
**N-Gr-3-48**	31.5	41.1	7.3	4.3	15.8	0.77
**N-Gr-10-48**	37.7	41.2	4.8	3.1	13.3	0.92

Table S2. XRD parameters obtained for the graphite and the N-doped graphene materials.

Carbon-Based Materials	2θ (°)	FWHM (°)	D (nm)	d (nm)	~n
**Graphite**	26.39	0.255	44.2	0.337	131
**N-Gr-3-24**	26.134	2.599	3.23	0.341	10 (9.5)
**N-Gr-10-24**	26.181	1.886	4.5	0.340	13
**N-Gr-3-48**	23.24	2.794	3.0	0.339	9 (8.8)
**N-Gr-10-48**	26.142	2.713	3.09	0.341	9 (9.1)

Where: θ—angle of diffraction; FWHM—full width of peaks at half maxima; D—crystallite size; d—interlayer spacing; n—number of graphene sheets.

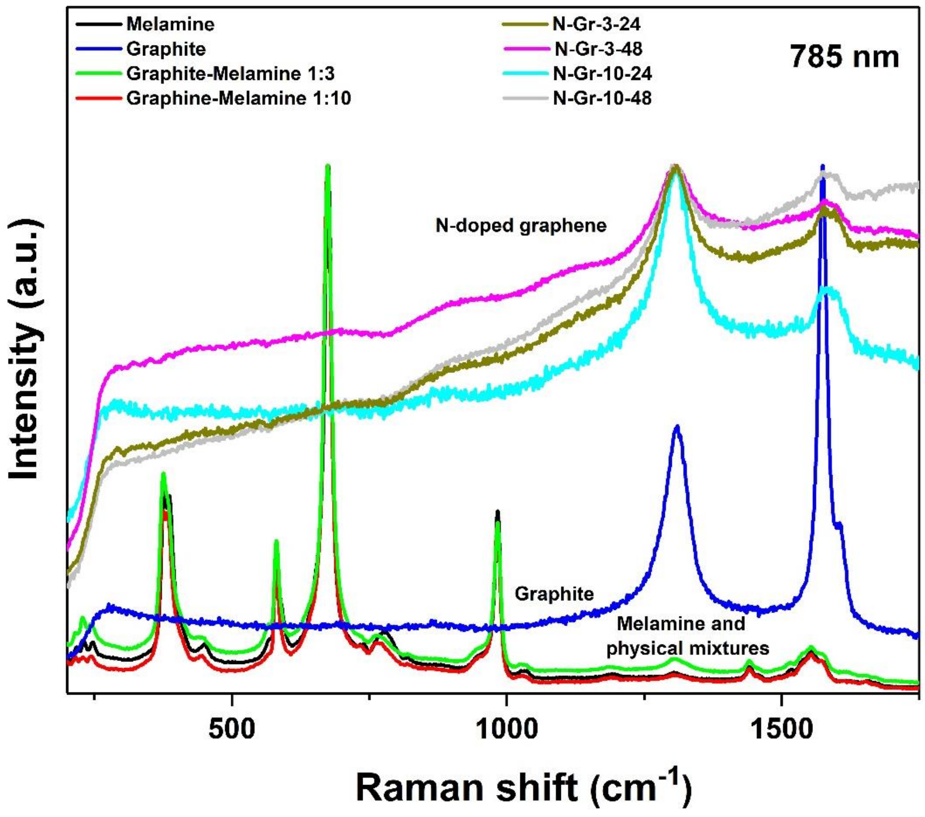

Figure S4. Raman spectra (excitation 785 nm) of the graphite, melamine, 1:3 and 1:10 graphite–melamine mixtures and N-doped graphene materials obtained by ball milling procedure.

Table S3. Raman parameters obtained for the N-doped graphene materials.

N-Doped Graphene	I_D/I_G	$I_{D'}/I_G$	I_{2D}/I_G	$I_D/I_{D'}$	N wt%
**Graphite**	0.15	***0.03***	***0.54***	5.83	–
**N-Gr-3-24**	2.02	***0.51***	***0.38***	3.94	9.61
**N-Gr-10-24**	1.58	***0.41***	***0.46***	3.90	8.34
**N-Gr-3-48**	2.00	***0.56***	***0.32***	3.54	13.1
**N-Gr-10-48**	1.95	***0.53***	***0.37***	3.70	11.4

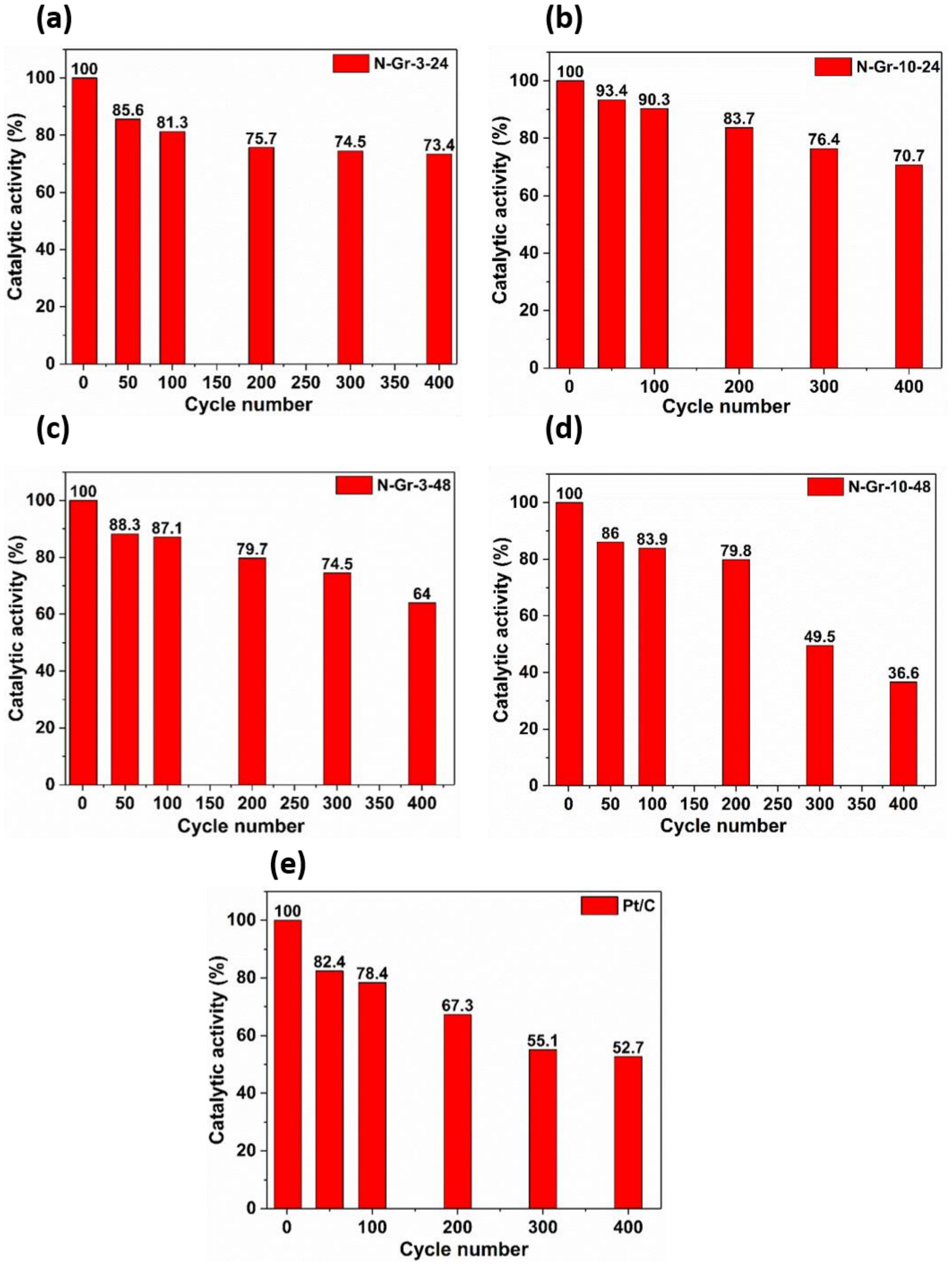

Figure S5. Evaluation of the catalytic activity retention (%) over 400 cycles of the (**a**–**d**) N-doped graphene/GC electrodes, respectively **(e)** Pt/C/GC electrode in O_2-saturated 1M NaOH solutions at a scan rate of 50 mV/s.

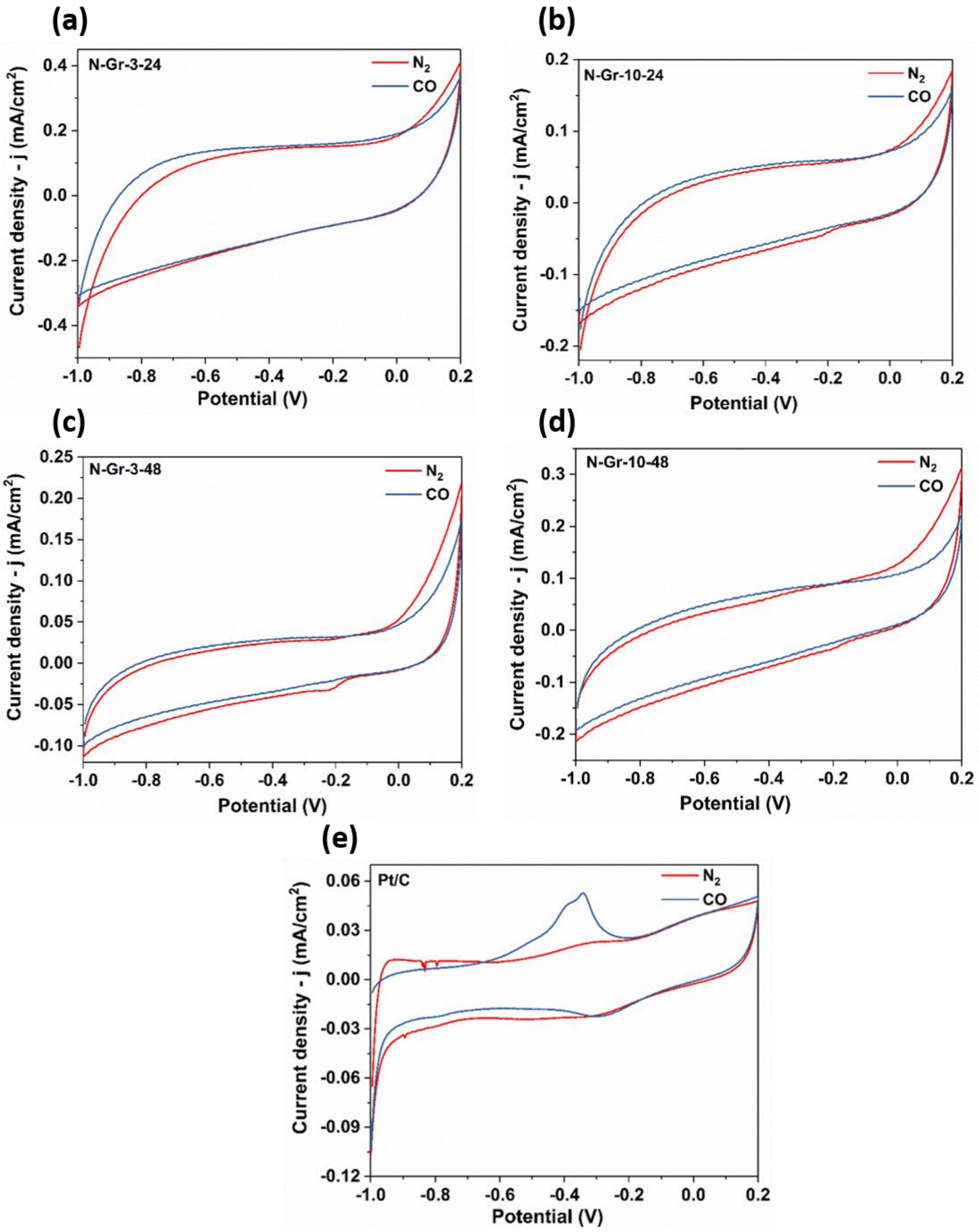

Figure S6. CO stripping voltammograms of (**a**–**d**) N-doped graphene/GC electrodes, respectively **(e)** Pt/C/GC electrode in 1M NaOH.

Table S4. Input and output variables used for ML investigations and the obtained prediction for a number of 4 electrons transferred during the ORR process.

Variables	N-Gr-3-24	N-Gr-10-24	N-Gr-3-48	N-Gr-10-48	Prediction
**Melamine (g)**	3	10	3	10	–
**Reaction time (h)**	24	24	48	48	*25*
**N-composition (weight%)**	9.61	8.34	13.1	11.4	*8.64*
**C-composition (weight%)**	80	81.3	72.5	80.2	–
**O-composition (weight%)**	9.31	9.43	12.98	7.34	–
**Pyridinic N (%)**	21.8	24.8	25.5	23.8	*19*
**Pyrrolic N (%)**	72.7	68.8	68.3	68.7	*72.9*
**Pyridinic N-O (%)**	5.5	6.3	6.1	7.5	–
**Csp2/Csp3**	0.94	0.97	0.77	0.92	*1.04*
**C-O (at%)**	5.8	6.2	4.3	3.1	*5.9*
**C=O (at%)**	9.8	10.5	15.8	13.3	*9.6*
**FWHM (°)**	2.599	1.886	2.794	2.713	–
**D (nm)**	3.23	4.5	3	3.09	–
**d (nm)**	0.341	0.34	0.339	0.341	–
**n**	9.5	13	8.8	9.1	–
I_D/I_G	2.02	1.58	2	1.95	–
$I_{D'}/I_G$	0.51	0.41	0.56	0.53	*0.44*
I_{2D}/I_G	0.38	0.46	0.32	0.37	*0.45*
$I_D/I_{D'}$	3.94	3.9	3.54	3.7	–
**G-band (cm^{-1})**	1593	1590	1596	1594	*1591*
Output (number of electrons)	*3.96*	*4.1*	*3.41*	*3.08*	
